# Quantitative anatomy of the primary ossification center of the squamous part of temporal bone in the human fetus

**DOI:** 10.1371/journal.pone.0295590

**Published:** 2023-12-07

**Authors:** Magdalena Grzonkowska, Mariusz Baumgart, Michał Kułakowski, Michał Szpinda

**Affiliations:** 1 Department of Normal Anatomy, The Ludwik Rydygier Collegium Medicum in Bydgoszcz, The Nicolaus Copernicus University in Toruń, Toruń, Poland; 2 Orthopaedic and Trauma Surgery Department, Independent Public Healthcare Center Rypin, Rypin, Poland; University of Szeged Institute of Biology: Szegedi Tudomanyegyetem Biologia Intezet, HUNGARY

## Abstract

Detailed numerical data about the development of primary ossification centers in human fetuses may influence both better evaluation and early detection of skeletal dysplasias, which are associated with delayed development and mineralization of ossification centers. To the best of our knowledge, this is the first report in the medical literature to morphometrically analyze the primary ossification center of the squamous part of temporal bone in human fetuses based on computed tomography imaging. The present study offers a precise quantitative foundation for ossification of the squamous part of temporal bone that may contribute to enhanced prenatal care and improved outcomes for fetuses with inherited cranial defects and skeletodysplasias. The examinations were carried out on 37 human fetuses of both sexes (16 males and 21 females) aged 18–30 weeks of gestation, which had been preserved in 10% neutral formalin solution. Using CT, digital image analysis software, 3D reconstruction and statistical methods, the size of the primary ossification center of the squamous part of temporal bone was evaluated. With neither sex nor laterality differences, the best-fit growth patterns for the primary ossification center of the squamous part of temporal bone was modelled by the linear function: *y* = −0.7270 + 0.7682 × age ± 1.256 for its vertical diameter, and the four-degree polynomial functions: *y* = 5.434 + 0.000019 × (age)^4^ ± 1.617 for its sagittal diameter, *y* = −4.086 + 0.00029 × (age)^4^ ± 2.230 for its projection surface area and *y* = −25.213 + 0.0004 × (age)^4^ ± 3.563 for its volume. The CT-based numerical data and growth patterns of the primary ossification center of the squamous part of temporal bone may serve as age-specific normative intervals of relevance for gynecologists, obstetricians, pediatricians and radiologists during screening ultrasound scans of fetuses. Our findings for the growing primary ossification center of the squamous part of temporal bone may be conducive in daily clinical practice, while ultrasonically monitoring normal fetal growth and screening for inherited cranial faults and skeletodysplasias.

## Introduction

The human skeletal system is one of the earliest and fastest developing systems during organogenesis. The calvaria includes the frontal, parietal, temporal, and occipital bones, which start to ossify between weeks 7 and 9 of gestation [1]. Their commensurate growth affords the normal morphology of the cranium and adequate brain development. During the fetal period the brain increases in size at a high rate and reaches approximately 80% of its final volume as early as at the end of year 2 of life [2].

During the prenatal development, the temporal bone is composed of the following five parts: squamous, styloid, mastoid, tympanic, and petrous [3]. As a typical calvarian bone, the squamous part of temporal bone–with the smaller anterosuperior part of the mastoid part of temporal bone–presents the intramembranous ossification. Contrariwise, the remaining parts of temporal bone–including the greater posteroinferior part of the mastoid part–refer to the cranial base, develop from the otic capsule, and ossify in an endochondral fashion [4,5]. In all, the temporal bone develops from ten primary ossification centers that are formed around traversing cranial nerves, the internal acoustic opening and the anterior, posterior and lateral semicircular canals [6].

The anatomy of the temporal bone is relatively complicated since it includes the organs of hearing and balance, and is traversed by numerous neurovascular structures, adequately encompassed by osseous canals and canaliculi. The temporal bone is the only cranial bone to articulate with the head of mandible, with the formation of the temporomandibular joint [7]. The temporal bone contributes to the formation of the middle and posterior cranial fossae, the medial and lateral walls of temporal fossa and the superior and posterior walls of infratemporal fossa [8].

Although the timing of ossification of each cranial bone is well-recognized, no morphometric measurements of the primary ossification center of the squamous part of temporal bone have been reported. This is the first report in the professional literature to morphometrically analyze the primary ossification center of the squamous part of temporal bone in human fetuses with the use of computed tomography imaging. It should be emphasized that the CT-based numerical data and growth patterns of the primary ossification center of the squamous part of temporal bone may effectively serve as age-specific normative intervals useful in daily clinical practice during screening ultrasound scans of fetuses.

In the present study we aimed:

- to determine normative age-specific reference intervals for linear, planar and volumetric parameters of the primary ossification center of the squamous part of temporal bone in human fetuses;
- to examine the possible laterality and sex differences for all analyzed parameters;
- to compute growth patterns for the analyzed parameters, expressed by best-matched mathematical models.

## Material and methods

### 1. Material

The study material comprised 37 human fetuses of Caucasian origin (16 males and 21 females) at the age of 18 to 30 weeks of gestation. The anatomical request of the study was granted by the Bioethics Committee of the Ludwik Rydygier Collegium Medicum in Bydgoszcz of the Nicolaus Copernicus University in Toruń (KB 275/2021). The fetuses were obtained from spontaneous miscarriages after parents expressed their written consent to use the fetuses in medical research. The fetuses were collected before the year 2000 and remain part of the fetal collection of the Department of Anatomy of the Ludwik Rydygier Collegium Medicum in Bydgoszcz of the Nicolaus Copernicus University in Toruń. All research actions were done in accordance with legal procedures in Poland and the program Donation Corpse for both adults and fetuses. Furthermore, this study was performed in line with the principles of the Declaration of Helsinki.

All morphometric examinations were carried out between the 1st of April 2022 and 31st of December 2022 at the Department of Anatomy of the Ludwik Rydygier Collegium Medicum in Bydgoszcz of the Nicolaus Copernicus University in Toruń. Each fetus was uniquely marked, so as to be unequivocally identifiable both during and after the data collection process. Based on evaluation of their explicit morphology and clinical records, fetuses with conspicuous congenital defects or intrauterine growth retardation were excluded from the study. All specimens with neither internal nor external visible morphological malformations were identified as normal and included in the morphometric study. It should be emphasized that the fetuses studied did not display any developmental abnormalities of the musculoskeletal system. Fetal ages were determined from the crown–rump length and the known date of the beginning of the last maternal menstrual period. The fetuses studied could not suffer from growth retardation as the correlation between the gestational age based on the crown–rump length (CRL) and that calculated using the last menstruation reached R = 0.98 (p < 0.001). Table 1 presents the characteristics of the study group, including age, number, and sex of the fetuses examined.

**Table 1:** Age, number and sex of the fetuses studied.

Gestational age (weeks)	Crown-rump length (mm)				Number	Sex	
					of fetuses		
	Mean	SD	Min.	Max.	N	♂	♀
18	133.33	5.77	130.00	140.00	3	1	2
19	146.50	2.89	143.00	150.00	4	2	2
20	161.00	2.71	159.00	165.00	4	2	2
21	173.67	2.31	171.00	175.00	3	2	1
22	184.67	1.53	183.00	186.00	3	1	2
23	198.67	2.89	197.00	202.00	3	1	2
24	208.00	3.56	205.00	213.00	4	1	3
25	214.00	–	214.00	214.00	1	0	1
26	229.00	5.66	225.00	233.00	2	1	1
27	240.33	1.15	239.00	241.00	3	3	0
28	249.50	0.71	249.00	250.00	2	0	2
29	253.00	0.00	253.00	253.00	2	0	2
30	262.67	0.58	262.00	263.00	3	2	1
Total					37	16	21

### 2. Methods

**Fig 1:**
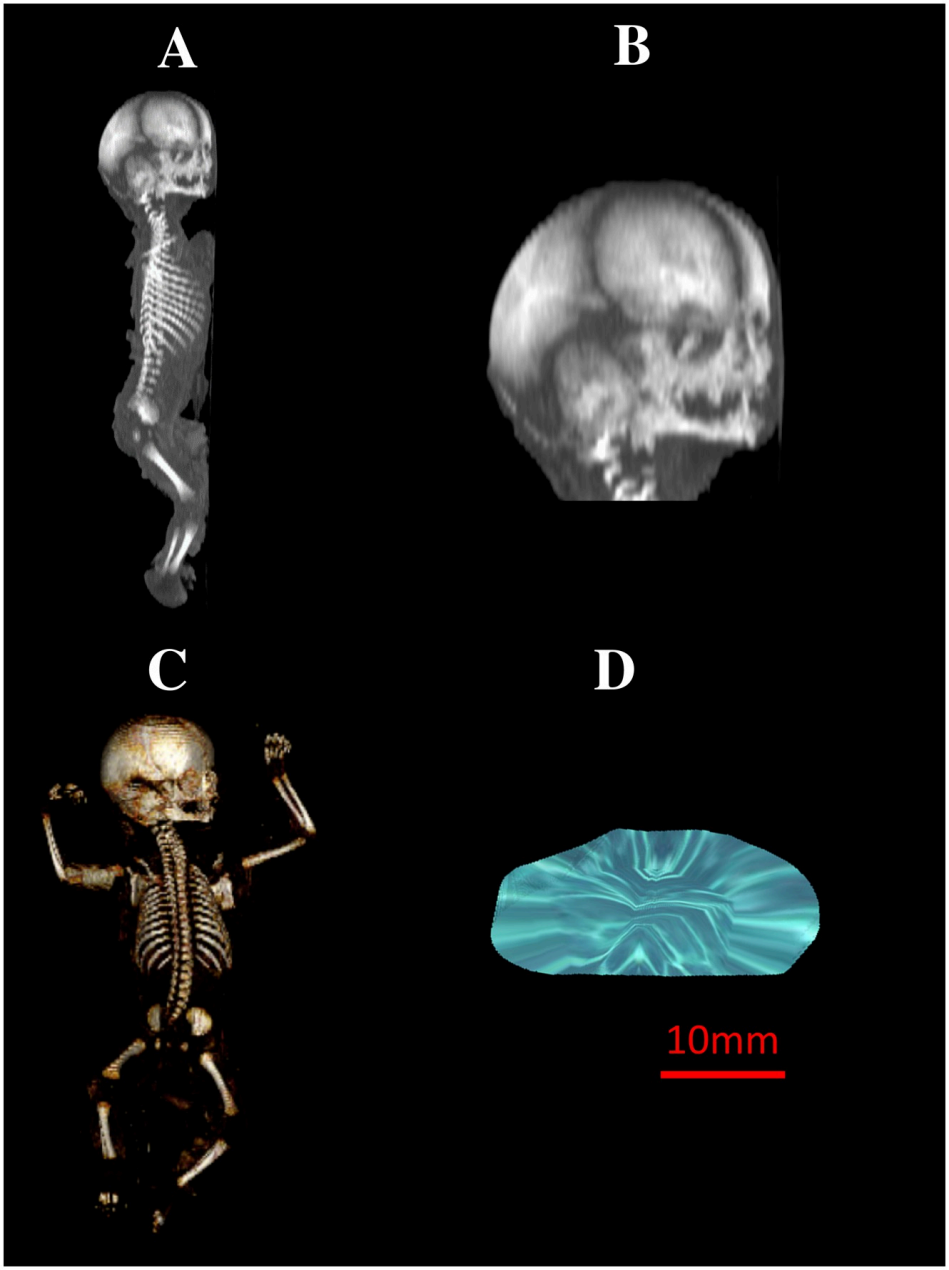
A female human fetus aged 22 weeks in the sagittal projection (A, B), its skeletal reconstruction in the frontal projection (C), 3D reconstruction of the primary ossification center of the squamous part of temporal bone (D) using Osirix 3.9 MD.

Using the Siemens-Biograph 128 mCT scanner (Siemens Healthcare GmbH, Erlangen, Germany) located at the Department of Positron Emission Tomography and Molecular Imaging (Oncology Center, the Ludwik Rydygier Collegium Medicum in Bydgoszcz, the Nicolaus Copernicus University in Toruń, Poland), scans of fetuses in DICOM format were acquired at 0.4 mm intervals (Fig 1).

**Fig 2:**
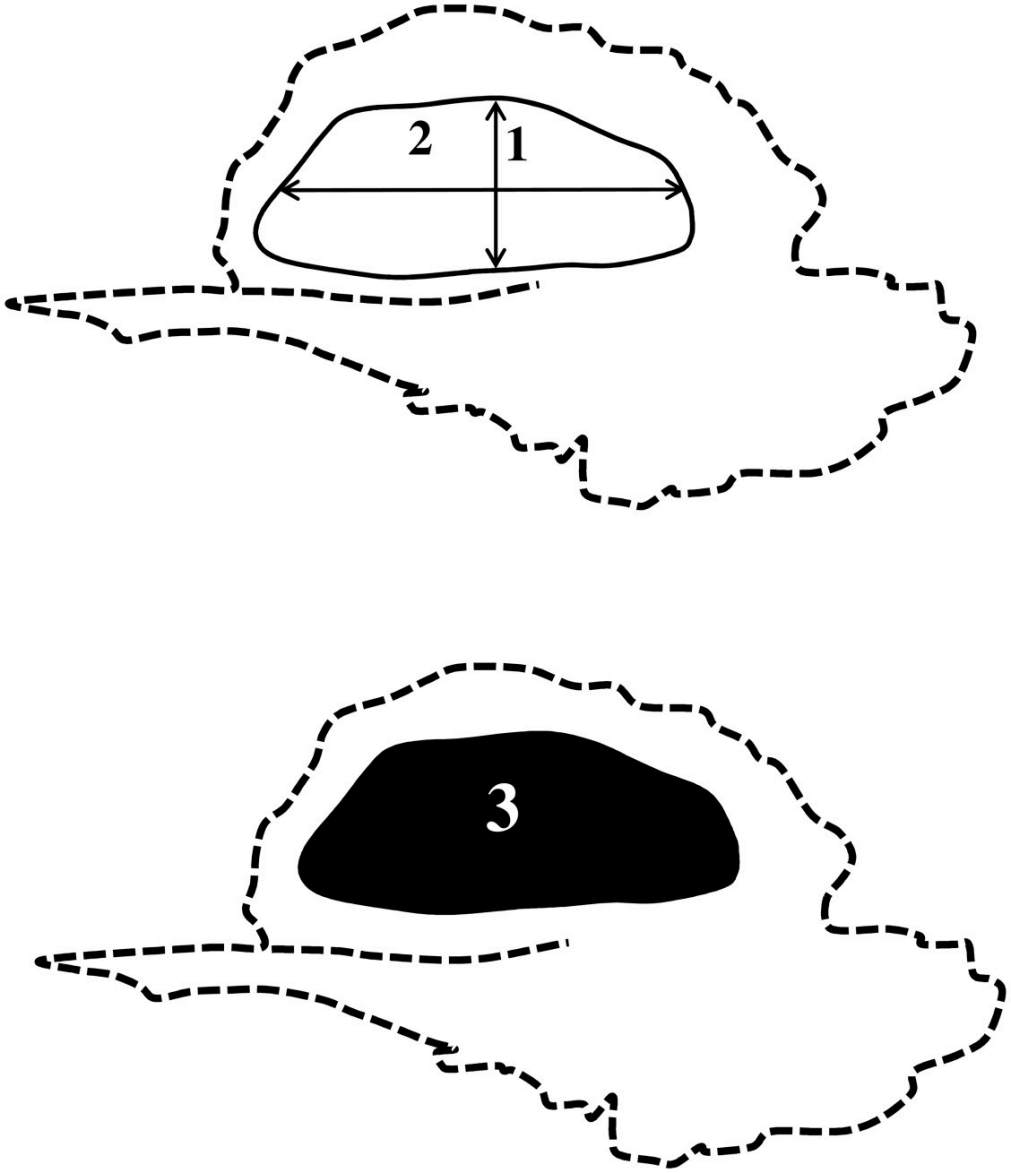
Measurement scheme of the primary ossification center of the squamous part of temporal bone: 1–vertical diameter, 2–sagittal diameter, 3–projection surface area.

The gray scale of the obtained CT images expressed in Hounsfield units (HU) varied from −275 to −134 for minimum, and from +1165 to +1558 for maximum. Therefore, the window width (WW) varied from 1.404 to 1.692, and the window level (WL) varied from +463 to +712. The specifics of the imaging protocol were as follows: mAs– 60, kV– 80, pitch– 0.35, FoV– 180, rot. time– 0.5 sec., while those of the CT data were: slice thickness– 0.4 mm, image increment– 0.6 mm, and kernel–B45 f-medium. Measurements of the squamous part of temporal bone were executed in a specific sequence (Fig 2).

In each fetus, the assessment of linear parameters, projection surface area and volume of the primary ossification center of the squamous part of temporal bone was carried out. Of note, the contours of both the squamous part of temporal bone and its primary ossification center were already evidently discernible [9,10].

On either side measurements of the primary ossification center of the squamous part of temporal bone included:

1. vertical diameter, based on the determined distance between its upper and lower borderlines in the sagittal plane (Fig 2);
2. sagittal diameter, based on the determined distance between its proximal and distal borderlines in the sagittal plane (Fig 2);
3. projection surface area, based on its determined contour in the sagittal plane (Fig 2); and
4. volume, calculated using advanced diagnostic imaging tools for 3D reconstruction, taking into account position and the absorption of radiation by bone (Fig 1C).

### 3. Statistical analysis

In this study, we used the Statistica 12.5 and PQStat 1.6.2 programs to analyze all individual numerical data. All numerical data underwent statistical analysis, which involved checking the distribution of variables using the Shapiro-Wilk (W) test and verifying the homogeneity of variance using Fisher’s test. To compare means, we employed Student’s t test for dependent (left-right) and independent (male-female) variables, followed by one-way analysis of variance and Tukey’s test for post-hoc analysis. In case with no similarity in variance, we used the non-parametric Kruskal-Wallis test. To identify the growth patterns of the analyzed parameters, we performed linear and curvilinear regression analysis, and finally we evaluated the match between the estimated curves and measurement results using the coefficient of determination (R^2^). Differences were considered statistically significant at p < 0.05. Furthermore, we assessed the relationships between variables using the Pearson correlation coefficient (R).

Each measurement was performed three times under the same conditions but at different times, and then averaged. As shown in Table 2, the intra-class correlation coefficients (ICC) were statistically significant (p < 0.001) and demonstrated excellent reproducibility.

**Table 2:** Intra-class correlation coefficients (ICC) values for inter-observer results.

Parameter	ICC
Right vertical diameter	0.998*
Left vertical diameter	0.997*
Right sagittal diameter	0.995*
Left sagittal diameter	0.996*
Left projection surface area	0.996*
Right projection surface area	0.996*
Right volume	0.998*
Left volume	0.997*

Intra-class correlation coefficients marked with * are statistically significant at p < 0.0001.

## Results

Tables 3 and 4 present the mean values and standard deviations of the analyzed parameters for the primary ossification center of the squamous part of temporal bone in human fetuses aged 18–30 weeks of gestation.

**Table 3:** Vertical and sagittal diameters, projection surface area and volume of the ossification center of the right squamous part of temporal bone in human fetuses.

Gestational age (weeks)	Number of fetuses	Ossification center of the right squamous part of temporal bone							
		vertical diameter (mm)		sagittal diameter (mm)		projection surface area (mm^2^)		volume (mm^3^)	
		Mean	SD	Mean	SD	Mean	SD	Mean	SD
18	3	7.00	0.46	8.21	0.01	34.10	10.06	37.23	11.23
19	4	7.57	0.17	8.73	0.41	44.30	3.06	50.19	3.97
20	4	8.47	0.09	9.60	0.17	54.47	1.53	64.42	2.05
21	3	8.98	0.17	10.05	0.12	60.49	1.80	72.20	2.41
22	3	9.39	0.12	10.22	0.09	64.28	1.33	77.57	1.92
23	3	10.00	0.26	10.50	0.12	69.40	1.10	84.91	2.34
24	4	10.72	0.25	10.31	0.54	74.73	1.80	92.10	2.35
25	1	11.50	–	10.21	–	78.67	–	98.34	–
26	2	11.79	0.13	11.87	1.64	93.83	13.97	119.69	18.48
27	3	13.87	0.57	14.76	1.42	137.58	18.44	180.84	25.68
28	2	14.35	0.21	18.03	2.55	169.05	20.70	234.65	37.04
29	2	14.93	0.52	18.98	0.21	194.38	2.44	275.10	13.08
30	3	16.73	0.95	23.33	3.73	263.11	57.47	390.58	87.37

**Table 4:** Vertical and sagittal diameters, projection surface area and volume of the ossification center of the left squamous part of temporal bone in human fetuses.

Gestational age (weeks)	Number of fetuses	Ossification center of the left squamous part of temporal bone							
		vertical diameter (mm)		sagittal diameter (mm)		projection surface area (mm^2^)		volume (mm^3^)	
		Mean	SD	Mean	SD	Mean	SD	Mean	SD
18	3	6.90	0.45	8.01	0.01	33.29	9.82	36.86	11.12
19	4	7.46	0.17	8.52	0.40	43.26	2.99	49.70	3.93
20	4	8.35	0.09	9.37	0.17	53.19	1.49	63.71	1.89
21	3	8.85	0.17	9.73	0.14	59.42	2.22	71.15	2.38
22	3	9.25	0.12	10.00	0.08	63.61	1.32	76.44	1.89
23	3	9.89	0.28	10.13	0.07	68.67	1.09	83.94	2.51
24	4	10.61	0.25	10.44	0.14	73.95	1.78	91.19	2.32
25	1	11.39	–	10.60	–	77.85	–	97.36	–
26	2	11.67	0.13	11.80	1.55	92.86	13.83	118.51	18.29
27	3	13.74	0.56	14.62	1.41	136.22	18.31	178.98	25.37
28	2	14.21	0.21	17.36	1.82	167.41	20.50	232.07	36.63
29	2	14.78	0.52	19.29	0.49	192.49	2.42	272.08	12.93
30	3	16.57	0.94	23.11	3.69	260.56	56.91	386.29	86.41

The statistical analysis revealed no significant differences in sex and laterality, and so we calculated one growth curve for each analyzed parameter.

The vertical diameter of the primary ossification center of the squamous part of temporal bone displayed a commensurate growth with gestational age. Contrariwise, the sagittal diameter, projection surface area, and volume increased with age expressed in weeks in accordance with the fourth-degree polynomial functions.

**Fig 3:**
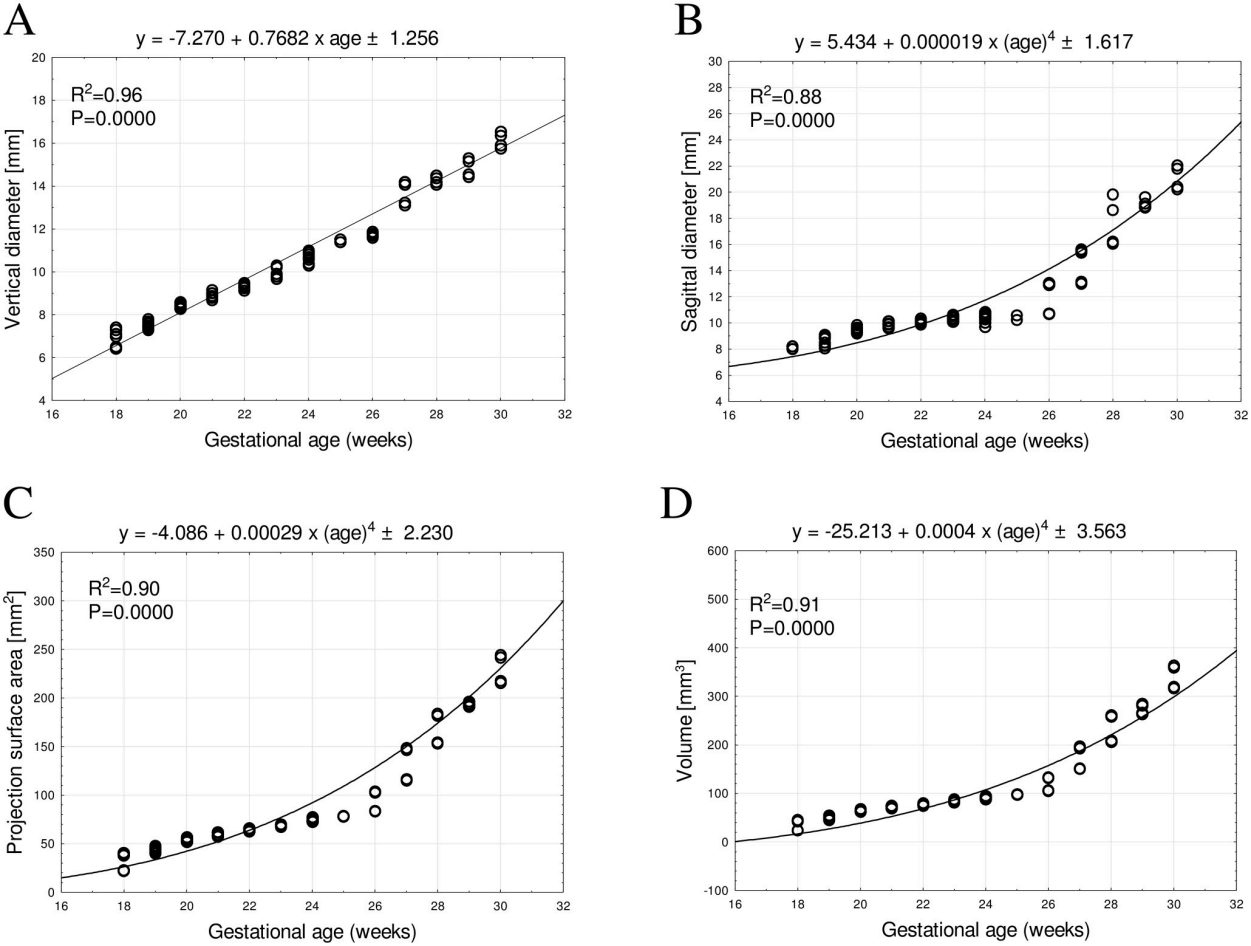
Regression lines for vertical diameter (A), sagittal diameter (B), projection surface area (C), and volume (D) of the primary ossification center of the squamous part of temporal bone.

At the gestational age range of 18–30 weeks, the mean vertical diameter of the primary ossification center of the squamous part of temporal bone ranged from 7.00 ± 0.46 mm to 16.73 ± 0.95 mm on the right, and from 6.90 ± 0.45 mm to 16.57 ± 0.94 mm on the left. The growth pattern followed the linear function of gestational age: *y* = −0.7270 + 0.7682 × age ± 1.256, with an R^2^ value of 0.96 (Fig 3A).

The mean sagittal diameter of the primary ossification center of the squamous part of temporal bone between weeks 18 and 30 ranged from 8.21 ± 0.01 mm to 23.33 ± 3.73 mm on the right, and from 8.01 ± 0.01 mm to 23.11 ± 3.69 mm on the left. The growth pattern produced the four-degree polynomial function: *y* = 5.434 + 0.000019 × (age)^4^ ± 1.617, with an R^2^ value of 0.88 (Fig 3B).

The mean projection surface area of the primary ossification center of the squamous part of temporal bone between weeks 18 and 30 increased from 34.10 ± 10.06 mm^2^ to 263.11 ± 57.47 mm^2^ on the right, and from 33.29 ± 9.82 mm^2^ to 260.56 ± 56.91 mm^2^ on the left, according to the four-degree polynomial function: *y* = −4.086 + 0.00029 × (age)^4^ ± 2.230, with an R^2^ value of 0.90 (Fig 3C).

Finally, between weeks 18 and 30 the mean volume of the primary ossification center of the squamous part of temporal bone grew from 37.23 ± 11.23 mm^3^ to 390.58 ± 87.37 mm^3^ on the right, and from 36.86 ± 11.22 mm^3^ to 386.29 ± 86.41 mm^3^ on the left. Such an increase followed the four-degree polynomial function of age: *y* = −25.213 + 0.0004 × (age)^4^ ± 3.563, with an R^2^ value of 0.91 (Fig 3D).

## Discussion

The discussion section is consecutively concentrated on the development of the temporal bone, the size of the primary ossification center of the squamous part of temporal bone and potential significance of the CT-based numerical data and growth patterns of this primary ossification center.

### 1. Development of the temporal bone

CT-based quantitative analyses of primary ossification centers of various cranial bones in the human fetus still remain limited due to a shortage of fetal material [11]. It should be emphasized that precise morphometric data of the primary ossification center of the squamous part of temporal bone in human fetuses may be valuable for the early detection of conspicuous developmental defects.

The ossification of the temporal bone initiates around 7 to 8 weeks of gestation through a combination of intramembranous and endochondral types of ossification. Specifically, one primary ossification center appears in the zygomatic process of temporal bone, while four primary ossification centers emerge in the tympanic part of temporal bone between weeks 11 and 12 of gestation [4,12]. Both the squamous and tympanic parts of temporal bone develop through intramembranous ossification and subsequently fuse with each other after birth. On the other hand, the remaining parts of the temporal bone, i.e. petrous and mastoid ones, contribute to the cranial base, and so develop through endochondral ossification [4].

The development of the tympanic cavity begins with the formation of air cells located within the petrous and mastoid parts of temporal bone. The tympanic cavity reaches its adult size by week 37 of gestation [6]. By month 9 of gestation, the bony labyrinth, the three auditory ossicles: malleus incus and stapes, and, the tympanic ring all achieve the adult size and do not increase after birth [13].

During the mid-fetal development, imaging examinations allow to identify both the squamous and petrous parts of temporal bone. According to Nemzek et al. [6], the squamous part of temporal bone with its zygomatic process may be identifiable in CT imaging as early as week 17 of gestation. Thus, in the present study based on fetuses at the gestational age of 18–30 weeks, CT imaging allowed for an accurate visualization of the primary ossification center of the squamous part of temporal bone in all the fetuses included.

It is noteworthy that the mastoid part of temporal bone does not have its own primary ossification center and successively develops from the petrous part of temporal bone [6,7,12]. At the age of month 9 of gestation, only three primary ossification centers are distinguishable in the temporal bone that start to aggregate [14].

During the postnatal period, both the shape and proportions of the mastoid, squamous and tympanic parts of temporal bone undergo a substantial evolution, with only the petrous part of temporal bone not being reorganized [13].

The normal development of the temporal bone determines the intracranial outflow of venous blood from the central nervous system during the fetal period, which is mainly due to the petrosquamous sinus. Before the internal jugular vein is formed, the petrosquamous sinus connects the dural venous sinuses to the retromandibular vein [15,16]. The petrosquamous sinus passes between the squamous and petrous parts of temporal bone. On the adulthood, only parts of the petrosquamous sinus persist [17]. After the internal jugular vein becomes the main venous drainage of the dural venous sinuses, the petrosquamous sinus will gradually be reduced to a minute emissary vein that interconnects the transverse sinus with the anterior division of retromandibular vein [18]. The petrosquamous sinus may be found in infancy and early childhood, with associated bony grooves or canals within the petrosquamous suture. However, the course of the petrosquamous sinus is extremely variable. The petrosquamous suture is present in the 9-month-old fetus, and after birth. Failure of the petrosquamous suture to close may lead to a dehiscence, which considerably increases predispositions to meningitis and cerebral infections [14].

### 2. Size of the primary ossification center of squamous part of temporal bone

This paper is the first report in the professional literature to quantitatively analyze the primary ossification center of the squamous part of temporal bone in human fetuses using CT and mathematical models describing its growth patterns.

Pryse-Davis et al. [19] found the advanced development of primary ossification centers to be faster in female fetuses. Furthermore, delayed ossification occurred more often in fetuses characterized by a small size and in the fetuses of multiparas. With the use of CT, Morimoto et al. [11] examined formalin-fixed fetuses, so as to study changes in the shape of cranial bones. These authors demonstrated a lack of sexual dimorphism in the fetal cranium. This finding remains in line with our results, since we did not find any sex differences in respect to the squamous part of temporal bone in human fetuses.

Mandarim-de-Lacerda and Alves [20] weighed each cranial bone in human fetuses. These authors found the viscerocranial bones like the vomer, palatine bone, mandible and maxilla to grow in a manner different to that of the neurocranial bones like the sphenoid, ethmoid, frontal, occipital, parietal and temporal bones. The growth of neurocranial bones proved to be much faster than that of viscerocranial bones. Furthermore, the fastest growth in the neurocranium referred to the calvarian bones.

In the present study including fetuses aged 18–30 weeks, the primary ossification center of the squamous part of temporal bone turned out to proportionately increase in its vertical diameter, following the linear function: *y* = −0.7270 + 0.7682 × age ± 1.256. The three remaining parameters of the primary ossification center of the squamous part of temporal bone modelled the fourth-degree polynomial functions: *y* = 5.434 + 0.000019 × (age)^4^ ± 1.617 for sagittal diameter, *y* = −4.086 + 0.00029 × (age)^4^ ± 2.230 for projection surface area and *y* = −25.213 + 0.0004 × (age)^4^ ±3.563 for volume.

After reviewing the professional literature, we managed to find only our previous articles concerning the quantitative analysis based on CT imaging with reference to primary ossification centers of the parietal, occipital and frontal bones in the human fetus. The primary ossification center of parietal bone grew in accordance with the fourth-degree polynomial functions: y = 21.746 + 0.000025 × (age)^4^ ± 1.256 for vertical diameter and y = 296.984 + 0.001 × (age)^4^ ± 6.971 for volume. Simultaneously, its sagittal diameter followed the quadratic function y = 16.322 + 0.0347 × (age)2 ± 1.323, while its projection surface area modelled the cubic function *y* = 284.1895 + 0.051 × (age)^3^ ± 0.490. The primary ossification center of squamous part of occipital bone grew commensurately in its vertical diameter: *y* = −6.462 + 1.109 × age ± 0.636 on the right, and *y* = −9.395 + 1.243 × age ± 0.577 on the left. The transverse diameters of the supraoccipital and interparietal parts as well as projection surface area of the primary ossification center of squamous part of occipital bone followed the natural logarithmic functions: *y* = −98.232 + 39.663 × *ln*(*age*) ± 0.721, *y* = −79.903 + 32.107 × *ln*(*age*) ± 0.974, and *y* = −3062.89 + 1108.98 × *ln*(*age*) ± 29.476, respectively. The volumetric growth of the primary ossification center of squamous part of occipital bone increased parabolically, following the quadratic function: *y* = −330.105 + 1.554 × (*age*)^2^ ± 32.559 [21]. The primary ossification center of squamous part of frontal bone grew parabolically in its vertical diameter, projection surface area and volume that was expressed by the following quadratic functions: *y* = 13.756 + 0.021 × (*age*)^2^ ± 0.024, *y* = 38.285 + 0.889 × (*age*)^2^ ± 0.034, and *y* = −90.020 + 1.375 × (*age*)^2^ ± 11.441, respectively. However, its transverse diameter increased in a proportionate fashion, following the linear function: *y* = 0.956 + 0.956 × *age* ± 0.823 [22].

There are no reports in the medical literature in terms of dimensions of the primary ossification center of the squamous part of temporal bone in human fetuses, which precludes a more comprehensive discussion on this topic.

### 3. Potential significance of the CT-based numerical data and growth patterns of the primary ossification center of the squamous part of temporal bone

Beyond doubt, knowledge about the growth of individual cranial bones in human fetuses may be useful in such numerous fields as gynecology, obstetrics, pediatrics, radiology, orthodontics, orthopedics, reconstructive surgery, anatomy and anthropology [20].

In fact, the present study of the ossification process of the squamous part of temporal bone is based on CT imaging criteria that is not routinely used in everyday clinical practice for fetal evaluation. However, the CT-based numerical data and growth patterns of the primary ossification center of the squamous part of temporal bone may serve as age-specific normative intervals. Because of this, our findings could effectively translate into a commonly used imaging technique such as ultrasound and may still remain of relevance for gynecologists, obstetricians, pediatricians and radiologists during screening ultrasound scans of fetuses.

It should be emphasized that the fetal monitoring and early detection of skeletal abnormalities are feasible due to both high-resolution ultrasonographic and CT examinations that allow for a precise evaluation of primary ossification centers in the fetus. Even though CT imaging is not usually used in clinical practice for fetal evaluation, in case of congenital defects of the temporal bone, it is preferable. CT is also the initial imaging method for the assessment of disorders of the temporal bone in most children and adults. The high-resolution and ultrathin CT slices provide excellent imaging of bony structures with the details of anatomical air spaces, making it an excellent tool for evaluating the structure of the pneumatized temporal bone. MRI is a complementary method in the diagnostics and subsequent planning of surgical treatment [3]. The abnormal development of the temporal bone may significantly contribute to a permanent hearing loss, usually resulting in speech impairment. Thus, an early and accurate diagnosis is crucial. A loss of hearing due to developmental defects of the temporal bone may be isolated or associated with more complex genetic disorders, such as Treacher Collins or Alport syndromes [3]. Abnormalities of the temporal bone are often clinically separated into those of the external, middle and internal ears. The development of individual parts of the ear is autonomous that explains the occurrence of defects of the middle or external ears with a normally developed internal ear, and *vice versa*. These abnormalities may occur in the temporal bone without any impairment of function or risk to the health of an individual [23].

Technique innovations with consecutive advances in medicine have created the possibility of obtaining more precisely defined fetal images, which assist in the medical diagnosis and contribute to the genetic counseling offered to parents during the prenatal period. In order to acquire *in utero* images, the main three technologies are primarily used: three-dimensional ultrasound (3D–US), magnetic resonance imaging (MRI) and computed tomography (CT). Standard prenatal screenings first include ultrasound, which may be devoid of specificity when dealing with suspected skull abnormalities. The lower contrast resolution in three-dimensional ultrasound may lead to difficulties in interpreting the image at gray-scale boundaries. The quality of obtained images is directly related to the precision of the morphometric data [24–26]. The sensitivity of ultrasound examination in the diagnosis of skeletal abnormalities is limited and ranges between 40% and 60% [27]. In order to achieve the precise diagnosis of skeletodysplasias, the evaluation should however involve more precise diagnostic imaging techniques, such as CT scans or MRI [28–30]. MRI examinations provide the best image quality in the late stages of pregnancy due to the limited space for fetal movements. The main difference between images obtained by computed tomography (CT) and magnetic resonance imaging (MRI) lies in the quality of contrast between internal organs on the MRI images [24]. Victoria et al. [27] found prenatal MRI results to insignificantly contribute to the further characterization of skeletal abnormalities. This is primarily due to both the difficulty in displaying a three-dimensional image of a fetus and obtaining images along the main axis of individual bones.

There is little information in the professional literature about the quantitative anatomy of the fetal skeletal system at specific gestational weeks with the use of CT when compared to ultrasound examinations. Nevertheless, a significant advantage of the CT technique lies in its ability to visualize the examined structure from multiple sides and at any point in time without compromising image details. Computed tomography should solely be performed with appropriate indications, proper technical parameters and an understanding that fetuses and children are much more sensitive to radiation than adults. The benefits of low-dose fetal computed tomography may outweigh the relatively small–but still real–individual risk. Consequently, CT for diagnosing minor fetal bone abnormalities should not be used primarily in daily clinical practice. However, CT may extremely be conducive in clinically difficult cases, in which an ultrasonographic diagnosis of a severe or potentially lethal disease remains equivocal [27].

## Limitations of the study

The main limitation of the present study was a relatively narrow gestational age group, ranging from 18 to 30 weeks, and a small number (N = 37) of human fetuses.

## Conclusions

1. The morphometric characteristics of the primary ossification center of the squamous part of temporal bone reveal neither laterality nor sex differences.
2. With advanced gestational ages, the primary ossification center of the squamous part of temporal bone grows linearly in respect to its vertical diameter and according to fourth-degree polynomial functions in respect to its sagittal diameter, projection surface area and volume.
3. The CT-obtained morphometric data of the primary ossification center of the squamous part of temporal bone may be considered as age-specific reference intervals that are contributable to the diagnostics of congenital defects and the estimation of gestational ages.
4. Further research on the growth and morphometric characteristics of the primary ossification center of the squamous part of temporal bone is warranted to expand an understanding about its development and potential clinical significance.

## Supporting information

S1 Appendix (PDF)

## References

[1] Burgos-Flórez FJ, Gavilán-Alfonso ME, Garzón-Alvarado DA. Flat bones and sutures formation in the human cranial vault during prenatal development and infancy: A computational model. J Theor Biol. 2016;393(21):127–144. doi: 10.1016/j.jtbi.2016.01.006 26780653

[2] Pattisapu JV, Gegg ChA, Olavarria G, Johnson KK, Ruiz RL, Costello BJ. Craniosynostosis: diagnosis and surgical management Atlas Oral Maxillofac Surg Clin North Am. 2010;18(2):77–91. doi: 10.1016/j.cxom.2010.08.002 21036311

[3] Nada A, Agunbiade SA, Whitehead MT, Cousins JP, Ahsan H, Mahdi E. Cross-sectional imaging evaluation of congenital temporal bone anomalies: what each radiologist should know. Curr Probl Diagn Radiol. 2020;50(5):716–724. doi: 10.1067/j.cpradiol.2020.08.005 32951949

[4] Jin SW, Sim KB, Kim SD. Development and growth of the normal cranial vault: an embryologic review. J Korean Neurosurg Soc. 2016;59(3):192–196. doi: 10.3340/jkns.2016.59.3.192 27226848 PMC4877539

[5] Lupu G, Popescu D, Panus V, Popescu G. Ontogenetic landmarks of the organ of hearing in fetal age determination. Rom J Leg Med. 2010;2:129–132. doi: 10.4323/rjlm.2010.129

[6] Nemzek WR, Brodie HA, Hecht ST, Chong BW, Babcook CJ, Seibert JA. MR, CT, and plain film imaging of the developing skull base in fetal specimens. AJNR Am J Neuroradiol 2000;21(9):1699–1706. . 11039353 PMC8174876

[7] Chmielewski JJ. New terminologia anatomica: cranium and extracranial bones of the head. Folia Morphol. 2021; 80(3):477–486. doi: 10.5603/FM.a2019.0129 31802475

[8] Koesling S, Kunkel P, Schul T. Vascular anomalies, sutures and small canals of the temporal bone on axial CT. Eur J Radiol. 2005;54:335–343. doi: 10.1016/j.ejrad.2004.09.003 15899333

[9] Chano T, Matsumoto K, Ishizawa M, Morimoto S, Hukuda S, Okabe H, et al. Analysis of the presence of osteocalcin, S-100 protein, and proliferating cell nuclear antigen in cells of various types of osteosarcomas. Eur J Histochem. 1996;40:189–198. . 8922947

[10] Duarte WR, Shibata T, Takenaga K, Takahashi E, Kubota K, Ohya K, et al. S100A4: a novel negative regulator of mineralization and osteoblast differentiation. J Bone Miner Res. 2003;18:493–501. doi: 10.1359/jbmr.2003.18.3.493 12619934

[11] Morimoto N, Ogihara N, Katayama K, Shiota K. Three-dimensional ontogenetic shape changes in the human cranium during the fetal period. J Anat. 2008; 212:627–635. doi: 10.1111/j.1469-7580.2008.00884.x 18430090 PMC2409084

[12] Krmpotic-Nemanic J, Padovan I, Vinter I, Jalsovec D. Prenatal and postnatal development of the tympanic portion of the temporal bone. Ann Anat. 1999;181(6):593–595. doi: 10.1016/S0940-9602(99)80073-9 10609060

[13] Scheuer L, Black S. Developmental juvenile osteology. San Diego, CA: Elsevier Academic Press, 2000, pp 18–170.

[14] Boot GW. Development of the temporal bone. JAMA. 1910;55(7):563–565. doi: 10.1001/jama.1910.04330070017005

[15] Cheatle AH. The petrosquamosal sinus: its anatomy and pathological importance. J Laryngol Otol. 1990;15:13–23. doi: 10.1111/j.1469-7580.2006.00652.x

[16] Chell J. The squamoso-petrous sinus: a fetal remnant. J Anat. 1991;175: 269–271. 2050572 PMC1224486

[17] Ruiz DSM, Gailloud P, Yilmaz H, Perren F, Rathgeb JP, Rufenacht DA, et al. The petrosquamousal sinus in humans. J Anat. 2006;209:711–720. doi: 10.1111/j.1469-7580.2006.00652.x 17118059 PMC2049005

[18] Chauhan NS, Sharma YP, Bhagra T, Sud B. Persistence of multiple emissary veins of posterior fossa with unusual origin of left petrosquamosal sinus from mastoid emissary. Surg Radiol Anat. 2011;33(9):827–831. doi: 10.1007/s00276-011-0822-x 21603953

[19] Pryse-Davies J, Smitham JH, Napier KA. Factors influencing development of secondary ossification centers in fetuses and newborn. Arch Dis Child. 1974;49:425–431. doi: 10.1136/adc.49.6.425 4852004 PMC1648819

[20] Mandarim-de-Lacerda CA, Alves MU. Growth of the cranial bones in human fetuses (2nd and 3rd trimesters). Surg Radiol Anat. 1992;14:125–129. doi: 10.1007/BF01794887 1641736

[21] Grzonkowska M, Baumgart M, Badura M, Wiśniewski M, Szpinda M. Quantitative anatomy of the fused ossification center of the occipital squama in the human fetus. PLoS One 2021;3;16(2):e0247601. doi: 10.1371/journal.pone.0247601 33621236 PMC7901728

[22] Grzonkowska M, Baumgart M, Badura M, Wiśniewski M, Szpinda M. Morphometric study of the primary ossification center of the frontal squama in the human fetus. Surg Radiol Anat. 2020;42(7): 733–740, doi: 10.1007/s00276-020-02425-7 32025797 PMC7261738

[23] Romo LV, Casselman JW, Robson CD. Temporal bone: congenital anomalies. Head and Neck Imaging. Curtin HD, Som PM (eds.) 2003;pp 1109–1172.

[24] Werner H, dos Santos JRL, Fontes R, Daltro P, Gasparetto E, Marchiori E, et al. Additive manufacturing models of fetuses built from three-dimensional ultrasound, magnetic resonance imaging and computed tomography scan data. Ultrasound Obstet Gynecol. 2010;36(3): 355–361. doi: 10.1002/uog.7619 20205157

[25] Rosignoli L, Tonni G, Centini G. Cranila development in the first trimester: the use of 3D in the study of complex structures. Imaging Med 2010;2(3): 251–257.

[26] Tonni G, Grisolia G, Sepulveda W, Rizzo G. Chapter "Ultrasound Imaging of Early Embryonic and Fetal Development", in: The Continous Textbook of Women’s Medicine Series", Obstetrics Module, Vol. 18, Ultrasound in Obstetrics, Editors: Professor Katia Bilardo, Dr. Valentina Tsibizova, September 2023. ISSN: 1756-2228; 3843/GLOWM.419363. https://www.glowm.com/article/heading/vol-18-u%F2ltrasound-in-onstetrics-ultrasound-imaging-of-early-embryonic-and-fetal-development/id/419363.

[27] Victoria T, Epelman M, Coleman BG, Horii S, Oliver ER, Mahboubi S, et al. Low-dose fetal CT in the prenatal evaluation of skeletal dysplasias and other severe skeletal abnormalities. AJR Am J Roentgenol. 2013;200(5): 989–1000. doi: 10.2214/AJR.12.9722 23617480

[28] Vu HL, Panchal J, Parker EE, Levine NS, Francel P. The timing of physiologic closure of the metopic suture: a review of 159 patients using reconstructed 3D CT scans of the craniofacial region. J Craniofac Surg. 2001;12(6): 527–532. doi: 10.1097/00001665-200111000-00005 11711818

[29] Van Zalen-Sprock RM, Brons TJ, van Vugt JMG, van der Harten HJ, van Gejin HP. Ultrasonographic and radiologic visualization of the developing embryonic skeleton. Ultrasound Obstet Gynecol. 1997;9(6): 392–397. doi: 10.1046/j.1469-0705.1997.09060392.x 9239824

[30] Nikolova S, Toneva D, Georgiev I, Lazarov N. Digital radiomorphometric analysis of the frontal sinus and assessment of the relation between persistent metopic suture and frontal sinus development. Am J Phys Anthropol. 2018;165(33): 492–506. doi: 10.1002/ajpa.23375 29266191

