# Supplementary material for: Quantitative anatomy of the primary ossification center of the squamous part of temporal bone in the human fetus
Source: PLoS One. 2023 Dec 7;18(12):e0295590. doi: 10.1371/journal.pone.0295590 (PMC10703256; doi:10.1371/journal.pone.0295590)
Supplement: S1 Appendix — (PDF) [file pone.0295590.s001.pdf]

Number of fetuses	Sex	Crown-rump length (mm)	Gestational age (weeks)	Right vertical diameter (mm)	Left vertical diameter (mm)	Right sagittal diameter (mm)	Left sagittal diameter (mm)	Right projection surface area (mm^2)	Left projection surface area (mm^2)	Rigt volume (mm^3)	Left volume (mm^3)
1	F	130	18	6.5	6.41	8.2	8.01	22.53	22.00	24.33	24.09
2	M	130	18	7.1	7.00	8.2	8.01	39.01	38.09	42.52	42.10
3	F	140	18	7.4	7.29	8.22	8.03	40.75	39.80	44.83	44.39
4	F	143	19	7.4	7.29	8.25	8.06	40.90	39.94	45.81	45.36
5	M	146	19	7.48	7.37	8.52	8.32	42.70	41.69	48.25	47.77
6	F	147	19	7.6	7.49	9.02	8.81	45.93	44.85	51.90	51.39
7	M	150	19	7.8	7.69	9.12	8.91	47.66	46.54	54.81	54.27
8	F	159	20	8.4	8.28	9.42	9.20	53.02	51.77	62.56	61.94
9	M	160	20	8.4	8.28	9.52	9.30	53.58	52.32	63.22	62.60
10	M	160	20	8.51	8.39	9.62	9.39	54.85	53.56	64.72	64.08
11	F	165	20	8.58	8.46	9.82	9.59	56.45	55.12	67.18	66.20
12	F	171	21	8.8	8.67	9.92	9.61	58.49	57.11	69.60	68.59
13	M	175	21	9	8.87	10.12	9.69	61.02	59.59	72.62	71.56
14	M	175	21	9.14	9.01	10.12	9.88	61.97	61.55	74.37	73.29
15	M	183	22	9.26	9.13	10.16	9.91	63.03	62.38	75.64	74.54
16	M	185	22	9.4	9.26	10.18	10.05	64.11	63.44	77.58	76.45
17	F	186	22	9.5	9.36	10.32	10.05	65.69	65.00	79.48	78.33
18	F	197	23	9.8	9.66	10.38	10.07	68.16	67.44	82.47	81.27
19	F	197	23	9.9	9.80	10.52	10.10	69.78	69.05	85.13	84.29
20	M	202	23	10.3	10.20	10.61	10.21	70.26	69.53	87.12	86.26
21	M	205	24	10.4	10.30	10.71	10.27	73.22	72.46	89.33	88.45
22	M	206	24	10.67	10.56	10.81	10.41	73.77	73.00	91.79	90.88
23	M	208	24	10.8	10.69	9.71	10.50	74.63	73.85	92.22	91.31
24	F	213	24	11	10.89	10.01	10.60	77.28	76.47	95.05	94.11
25	F	214	25	11.5	11.39	10.21	10.60	78.67	77.85	98.34	97.36
26	M	225	26	11.7	11.58	10.71	10.70	83.96	83.08	106.62	105.57

27	F	233	26	11.88	11.76	13.03	12.89	103.71	102.63	132.75	131.44
28	M	239	27	13.22	13.09	13.13	12.99	116.30	115.08	151.19	149.69
29	M	241	27	14.2	14.06	15.53	15.38	147.75	146.32	195.03	193.11
30	M	241	27	14.2	14.06	15.63	15.48	148.70	147.26	196.29	194.13
31	F	249	28	14.2	14.06	16.23	16.07	154.41	152.92	208.46	206.17
32	F	250	28	14.5	14.36	19.83	18.65	183.69	181.91	260.84	257.97
33	F	253	29	14.56	14.42	18.83	18.94	192.65	190.78	265.85	262.93
34	F	253	29	15.3	15.15	19.13	19.64	196.10	194.20	284.35	281.22
35	F	262	30	15.9	15.75	20.43	20.23	217.64	215.53	319.93	316.42
36	M	263	30	16.53	16.37	22.03	21.82	243.98	241.62	363.54	359.54
37	M	263	30	17.76	17.59	27.54	27.27	327.70	324.53	488.28	482.91
